# SGLT2 inhibition alleviated hyperglycemia, glucose intolerance, and dumping syndrome-like symptoms in a patient with glycogen storage disease type Ia: a case report

**DOI:** 10.1186/s13256-020-02658-5

**Published:** 2021-02-16

**Authors:** Daisuke Katayama, Hiroo Baba, Takashige Kuwabara, Jun Kido, Hiroshi Mitsubuchi, Shirou Matsumoto, Kimitoshi Nakamura

**Affiliations:** 1 Department of Pediatrics, Kumamoto University Hospital, Honjo 1-1-1, Kumamoto City, Japan; 2 Department of Endocrinology, Beppu Hospital, Saifu 1-6-23, Dazaifu City, Japan; 3 Department of Nephrology, Graduate School of Medical Sciences, Kumamoto University, Honjo 1-1-1, Kumamoto City, Japan; 4 Department of Pediatrics, Graduate School of Medical Sciences, Kumamoto University, 1-1-1 Honjo, Kumamoto City, Kumamoto Prefecture 860-8556 Japan; 5 Division of Neonatology, Kumamoto University Hospital, Honjo 1-1-1, Kumamoto City, Japan

**Keywords:** GSD type Ia, Glucose intolerance, SGLT2 inhibitor

## Abstract

**Background:**

Glycogen storage disease (GSD) type Ia is a glycogenesis disorder with long-term complications such as hepatomegaly and renal dysfunction and is caused by congenital loss of glucose-6-phosphatase (G6Pase) expression. G6Pase is essential for the final step of gluconeogenesis and glycogenolysis, and its deficiency causes clinical hypoglycemia in the fasting state during infancy. Contrastingly, patients also show blood glucose trends and glucose intolerance similar to those in type II diabetes. Owing to the contrasting presentation of hypoglycemia with glucose intolerance, glucose control in patients remains a challenge, requiring management of both fasting hypoglycemia and post-prandial hyperglycemia.

**Case presentation:**

The patient was a 45-year old Asian (Japanese) woman who showed disease onset at 3 years of age, when hypoglycemia and hepatomegaly were observed, and GDS type Ia was diagnosed by the lack of G6Pase activity. Over the past 45 years, she presented hyperglycemia and dumping syndrome like symptoms (a feeling of fullness, even after eating just a small amount, abdominal cramping, nausea, sweating, flushing, or light-headedness and rapid heartbeat) at 2 hours after food intake. Her liver and kidney dysfunction also worsened over time. Treatment with exercise combined with a sodium-glucose co-transporter 2 inhibitor and an alpha glucosidase inhibitor alleviated her glucose intolerance and dumping syndrome-like symptoms, without increasing hypoglycemic events.

**Conclusion:**

This case suggests SGLT2 inhibitor as a promising candidate for treating glucose intolerance in GSD type Ia without worsening of hypoglycemia.

## Background

Glycogen storage disease (GSD) type Ia is an autosomal recessive disorder caused by dysfunction of the gene encoding glucose-6-phosphatase (G6Pase), a microsomal membrane component protein that is a critical enzyme in the final step of glycolysis and glycogenesis. G6Pase deficiency leads to hypoglycemia and accumulation of glycogen in several tissues, such as the muscle, kidney, and liver. Hypoglycemia is known to improve gradually because of G6Pase beta [1]. Contrastingly, patients also present glucose intolerance and dynamic plasma glucose levels, similar to those in type 2 diabetes, and parallel treatment of these contrasting conditions remains a challenge.

The sodium-glucose co-transporter 2 (SGLT2) inhibitor is a selective inhibitor that prevents glucose reabsorption by the proximal renal tube and corrects blood glucose levels to prevent glucose toxicity. Hypoglycemia is rare with the clinical usage of SGLT2 inhibitors because the other subtype of the inhibitor, SGLT1, continues to remain active and prevents extreme hypoglycemia. Several recent clinical trials indicated that SGLT2 inhibition is an effective strategy for diabetes mellitus and concluded that it (1) facilitates reduction of body weight and visceral fat, (2) prevents progressive kidney disease, and (3) improves liver function in non-alcoholic steatohepatitis (NASH) occurring with diabetes mellitus. All the complications mentioned above are also observed In GSD type Ia and a previous case report indicated that SGLT2 inhibitor improved diabetes mellitus in GSD type Ia [2].

In this context, we present here a case of successful alleviation GSD type Ia symptoms using a combination of exercise and an SGLT2 inhibitor.

## Case presentation

The patient was a 45-year-old Asian (Japanese) woman, who presented polycystic ovarian syndrome, hypertension, hyper-urinary acid, bladder stone, urinary stone, progressive renal dysfunction, and lipid abnormality. When she was 3 years old, she visited our hospital because of hypoglycemia, liver enzyme elevation, and hepatomegaly. She was diagnosed as having GSD type Ia based on the loss of G6Pase activity in the liver (< 0.3 µmol/minute/g tissue).

In the first stage of life (3 years to 6 years), she needed frequent feedings and a night time cornstarch regimen until 7 years of age. After 8 years of age, her hypoglycemia improved and her glucose levels could be maintained with cornstarch intake twice a day. However, she had several complications such as kidney stones and hepatic adenomas with hepatomegaly.

**Fig. 1.**
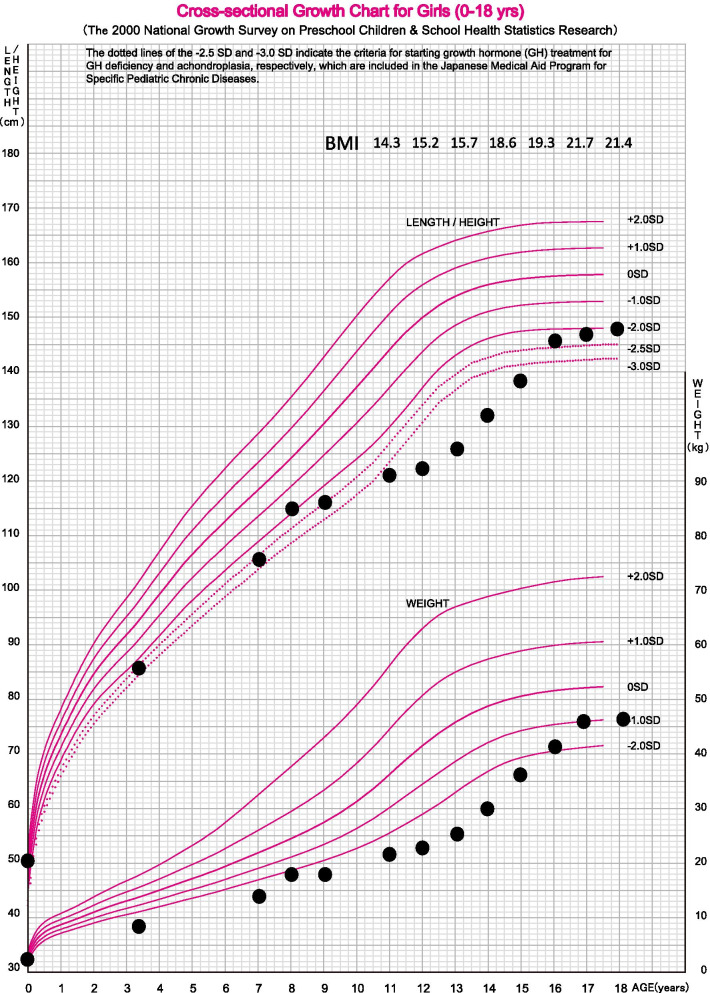
Growth curve

**Fig. 2.**
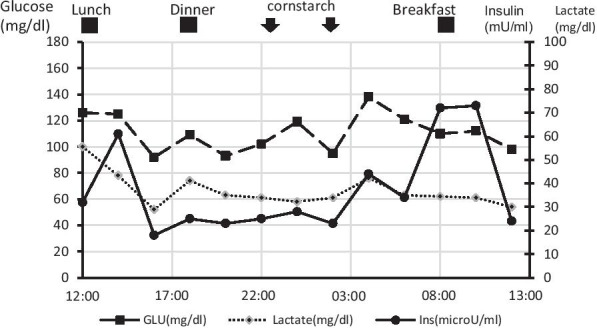
Daily blood sugar fluctuation

At 20 years of age, her BMI increased (to 24.2) and she presented hyperglycemia after food intake (Fig. 1). The blood glucose dynamics indicated type 2 diabetes mellitus as described in Fig. 2 [Fasting glucose: 123 mg/dl, homeostatic model assessment of insulin resistance (HOMA-IR) 9.23]. Therefore, cornstarch feeding at night was stopped.

**Fig. 3.**
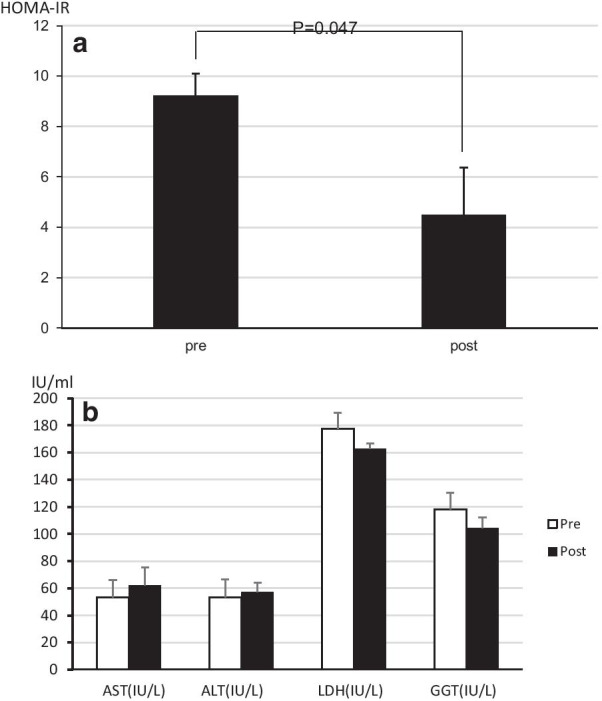
Changes in Laboratory tests before and after treatment

At 40 years of age, she presented hyperglycemia (200–260 mg/dl after feeding and dumping syndrome-like symptoms such as nausea, general fatigue, and dorsal pain). Moreover, the dumping syndrome-like symptoms worsened, resulting in poor quality of life. At first, she misunderstood that the complication was related to hypoglycemia and thus increased feeding, leading to an increase in body weight (maximum BMI was 26.4). Laboratory tests indicated elevated triglycerides (880 mg/dl) and abnormal liver function tests (Fig. 3). Magnetic resonance imaging (MRI) of the abdomen revealed multiple liver adenomas with diffuse steatosis. Investigation of diabetes showed an insulin level of 68 µIU/ml (normal range: ≦ 18.7 µIU/ml), with fasting blood glucose levels of 126 mg/dl (normal range: 70–114 mg/dl). HOMA IR was calculated as 22.1 (*N* < 2.5), indicating severe insulin resistance.

At first, she attempted lifestyle modifications such as a lower carbohydrate diet, elongation of feeding time, and exercise. However, her condition did not improve. To avoid severe hypoglycemia and improve liver function, she was experimentally treated with luseogliflozin hydrate additionally, an SGLT2 inhibitor, at a dose of 2.5 mg daily, after obtaining informed consent. After this intervention, she decreased her cornstarch intake, and her dumping syndrome-like symptoms disappeared completely. In addition, her body weight (BMI 21) and insulin resistance both decreased remarkably (HOMA-IR 4.49; Fig. 3).

## Discussion

GSD1 is characterized by severe fasting hypoglycemia because of the lack of G6Pase-α, but several studies have indicated that patients could demonstrate glucose production [3–5]. This recovery may be related to the widespread G6Pase-β expression in the body [6]. GSD-Ia children show hypoglycemia, but with age, their endogenous glucose production rate improves, starting from 50% of the normal in young GSD-Ia patients, reaching 67–100% of the normal in adult GSD-Ia patients [3–5]. Because muscle mass increases with age, forming 20% of the body weight in a newborn, 36% in adolescence, and 40–45% of adulthood [6], the muscle G6Pase- β/G6PT complex may be the source of some or all of the extra blood glucose. Upon undergoing such an age-related change in the muscle volume as well as visceral fat and due to fatty liver and increased BMI because of frequent feeding, patients show insulin resistance and their blood glucose level dynamics are similar to those in type 2 diabetes. The reason for glucose resistance and hyperglycemia in GSD type Ia is unclear, but seems to be caused by frequent feeding, night time feeding, raw cornstarch, body weight gain, liver dysfunction, and renal dysfunction, all of which could cause insulin resistance and abnormal endogenous glucose production. Frequent hypoglycemia could also induce (1) downregulation of insulin/glucagon ratio leading to the release of free fatty acids, and (2) stimulation of glycogenesis in the liver leading to increased malonyl Co A and inhibition of beta oxidation. Fatty liver in GSD type Ia is also thought to be caused by these mechanisms [7]. In contrast, excessive carbohydrate, which is needed to prevent hypoglycemia, is stored as glycogen in the liver, causing non-alcoholic fatty liver disease (NAFLD). It is reported that 69.8% of patients with NAFLD presented a glucose intolerance pattern [8]. In our patient, NAFLD/NASH since teenage and body weight gain were undoubtedly related to insulin resistance. Therefore, she was treated with an SGLT2 inhibitor, which could inhibit glucose reabsorption in the proximal renal tubule, release excessive glucose into urine and correct the plasma glucose levels. In addition, hypoglycemia is not expected because SGLT1 is still active and capable of preventing hypoglycemia. Preclinical studies indicate that the SGLT2 inhibitor could reduce insulin resistance, visceral fat, and body weight. A large clinical study in the UK (DAPA-HF study) indicated that the SGLT2 inhibitor could reduce the risk of cardiovascular death [9]. Another global clinical trial (DAPA-CKD Study, Phase III) indicated that an SGLT2 inhibitor (dapagliflozin) reduced the risk of a composite of a sustained decline in the estimated GFR of at least 50%, end-stage kidney disease, or death from renal or cardiovascular causes [10]. Further, A prospective, single-arm trial (LEAD trial) indicated that Luseogliflozin could be a novel promising agent for treating patients with T2DM and NAFLD [11].

In our case, the SGLT2 inhibitor showed good performance in suppressing the dumping syndrome-like symptoms mainly by alleviating hyperglycemia after feeding. In hypoglycemia, the SGLT2 inhibitor could reduce episodes of hypoglycemia and decrease between-meal eating, which may cause loss of body weight. Laboratory data related to NAFLD/NASH remained unchanged after intervention, but we consider that these may require a long-term treatment for improving.

## Conclusion

Our case indicates that the SGLT2 inhibitor may be a potential candidate medication for GSD type Ia with insulin resistance and diabetes.

## Data Availability

All data used during this study are included in this published article.

## Abbreviations

GSD: Glycogen storage disease type Ia
SGLT2: Sodium-glucose co-transporter 2
BMI: Body mass index
DM: Diabetes mellitus
NASH: Non-alcoholic steatohepatitis
NAFLD: Non-alcoholic fatty liver disease
G6Pase: Glucose-6 phosphatase
G6PT: Glucose-6-phosphate transporter
